# How to Disrupt Harmful Corporate Political Activity? Comment on "Corporate Political Activity: Taxonomies and Model of Corporate Influence on Public Policy"

**DOI:** 10.34172/ijhpm.9118

**Published:** 2025-12-02

**Authors:** Luc L. Hagenaars, Joreintje D. Mackenbach

**Affiliations:** ^1^Amsterdam Public Health, Amsterdam, The Netherlands.; ^2^Public & Occupational Health, Amsterdam UMC Location University of Amsterdam, Amsterdam, The Netherlands.; ^3^Epidemiology & Data Science, Amsterdam UMC Location Vrije Universiteit, Amsterdam, The Netherlands.; ^4^Upstream Team, Amsterdam UMC, Amsterdam, The Netherlands.

**Keywords:** Commercial Determinants of Health, Health Harming Industry, Policy Process, Public Health

## Abstract

Ulucanlar and colleagues’ model of corporate political activity by unhealthy commodity industries identifies cross-industry framing and actions that block public health policies. While the model helps to understand policy inertia around the prevention of commercially-driven diseases, it needs expansion into broader theories on policy change and framing to help understand how policy breakthroughs can be achieved. We substantiate this viewpoint by introducing four established policy theories, and by situating Ulucanlar and colleagues’ model within our application of the Punctuated Equilibrium Theory (PET), a theory that explicitly focuses on disruption of policy inertia. We outline how Ulucanlar and colleagues’ taxonomy can help investigate the strength of an industry’s policy monopoly and identify when and how an industry’s power might be crumbling. We further situate Ulucanlar and colleagues’ model within the concept of metaphorical framing, arguing that more robust grounding of the model in frameworks on morality can effectively challenge framing strategies of unhealthy commodity industries.

 In 2023, Ulucanlar and colleagues published their model and taxonomy of corporate political activities by unhealthy commodity industries.^1^ As to date, the article has been cited 40 times already, and the taxonomy has been applied to the tobacco, alcohol, food, and gambling industries, as well as industries such as pesticides, big tech, cannabis and transportation.^2^ This rapid citation track record indicates that the commercial determinants of health (CDoH) field is in need of good theory on corporate political practices, which have been identified as one of seven major commercial practices affecting health and equity.^3^ Whereas other corporate political activity models focused on single industries, Ulucanlar and colleagues qualitatively analysed papers that cover corporate political activities spanning the “big four” tobacco, alcohol, food and gambling industries. Their model details how corporate actors across these industries construct and present similar frames related to actors, problems and solutions, with a shared aim to avert the adoption of public health policies that may harm their interests.

 Ulucanlar and colleagues’ corporate political activity model is helpful because its cross-industry cutting focus reflects reality. Not only are frames and actions often similar,^3^ unhealthy commodity industries are partly driven by shared ownership. Fazzino et al recently demonstrated that major US tobacco companies owned leading US food companies between the 1980s and early 2000s, and that these food companies were more likely to introduce hyper-palatable foods into the food system compared to food companies that were not tobacco-owned.^4^ Similar deep ties are seen with tobacco and pharmaceutical companies,^5^ and ultra-processed food and weight-loss companies: Weight Watchers was owned by Kraft Foods, Slimfast by Unilever, and Jenny Craig by Nestlé.^6^

## Corporate Political Activities Likely to Differ Across Time and Space

 That is not to say that all unhealthy commodity industries deploy similar frames and actions at the same point in time. Rather, corporate political activities fluctuate in time and space based on the maturity and power of the industry,^7^ the salience of the issue in which an industry has an interest,^8^ and the cultural, political, and social contexts with which the industry interacts and in turn drives issue salience. For instance, the gambling and food industry now use similar arguments against regulatory policies as the tobacco industry did in the 1980s.^9,10^ These include that the evidence of health harms is inconclusive; that the problem lies elsewhere than in the product itself (eg, “problematic gamblers”); and that the industry “is part of the solution” and therefore a legitimate policy actor that can self-regulate, despite evident conflicts of interests. Compared to the gambling and food industries, the tobacco industry faced scientific and public scrutiny much earlier. As a result, it had to start pursuing tacit corporate political strategies that go beyond formal institutional arrangements. In contrast, the “issue salience” of the gambling and food industry practices is still relatively weak, explaining why it is still seen as relatively normal for these industries to influence policy either directly or through the funding of scientific research or “front group” civil society organizations.^8^

 Ulucanlar et al also highlight that corporate political activities take different shapes in non-democratic compared to democratic nations. But even within democratic nations, orporate political activities likely depend on cultural norms regarding the interaction between governments and industry partners in the policy process. For instance, in neocorporatist political systems—where formal institutions have traditionally allowed commercial actors to negotiate public policies with labour and government actors—there may be less need for “giving incentives” or “making threats”^1^ to influence the policy process than in “pluralist” political systems – which allow various interest groups to compete over the shape of public policies.^11^ The combination of the abovementioned temporal and spatial context with which unhealthy commodity industries interact determines what frames and actions are used when and where.

## Policy Process Theories

 Knowing how unhealthy commodity industries block public health policies, the onus of Ulucanlar and colleagues’ model, is necessary but not sufficient to bring about positive change. To overcome the policy inertia that plagues the CDoH, recent calls emphasize the importance for the CDoH field to engage more with theories and methods from the political science, macroeconomics, sociology of markets and other social sciences.^12-14^ “Policy process theories” are particularly relevant in the context of corporate-political activity, dozens have emerged over the past decades.^15^ We have depicted four of the more established theories in Table, highlighting their relevance to the CDoH. Any one theory captures an incomplete depiction of the policy process. Whereas the Punctuated Equilibrium Theory (PET)^16^ tries to explain how the institutional setting of an issue produces incrementalist policies with occasional “bursts” that disrupt the status quo, Kingdon’s Multiple Streams Framework (MSF) focuses on how “windows of opportunity” for policy change open when the way in which a problem is understood (problem stream), dynamics within the political arena (political stream), and the construction of policy solutions (policy stream) concur. The Advocacy Coalition Framework (ACF) can be used to analyse how coalitions pursue change or stability around a specific issue or policy. The Narrative Policy Framework (NPF) has the same dual usage but is set up to study how narratives shape policy.

**Table:** Elements of Established Policy Process Theories and Their Value for Studying Corporate-Political Activities of Health-Harming Industries

	**PET**	**MSF**	**ACF**	**NPF**
Scope and level of analysis	Focuses on why political systems are characterized by long periods of inertia and “small p policy” (incrementalism) and rare periods of “big P policy” (major reforms). It theorizes how shifts from stability to change concur with shifts in policy “ownership” from issue-specific policy subsystems (eg, finance, health, or trade departments) to the macropolitical system (eg, national parliaments), meaning that health-harming industry lobbyists must interact mostly with specialized policy subsystems if stability furthers their cause, or lobby political leaders if they need change (eg, to organize tax exemptions) or need to prevent change (eg, to prevent minimum unit prices for alcohol).	Focuses on policy choice under ambiguity. It has implicit focus on the political system with much emphasis on how actors couple the problem, political and policy “streams.” This refers to how society, politicians, and policy experts tend to live in separate streams, and assumes that windows of opportunity for policy change only open when these three streams collide. This makes it important for lobbyists to influence how society thinks about a problem, whether and how politicians relate to it, and what policy measures are considered by policy experts.	Focuses on interaction in advocacy coalitions aiming to change specific policy outcomes, and their learning from failures and successes. It has explicit focus on the resources (eg, money, networks, knowledge) and beliefs of coalitions. A focus on advocacy coalitions instead of the political system, allows for analysis of how industry lobbyists either establish coalitions themselves or prevent or fight existing coalitions whose goals and ideas misalign industry interests. Examples would include industry weaponization of patient organizations, or strategic fund allocation to drive apart coalitions whose deep core beliefs might align, but who might think differently about specific policy solutions.	Positions narratives as central for policy change, because they affect public opinion and policy agendas. Level: individual, coalitional, and societal. Through its focus on narratives, the NPF allows inquiry of how industry framing affects public opinion and how it can persuade individual politicians, and thereby change or maintain agendas, hypothesizing that narrative competition can lead to shifts in the policy agenda.
Underlying models of individual behaviour	Boundedly rational, particularly relating to attention. Assumes policy-makers cannot digest all information about the vast amount of issues that end up on their desk, so they ignore most and only prioritize some issues that align with their ideology. When policy stability aligns with the interests of health-harming industries, their lobbyists may have an incentive not to provide any information at all. When there is industry interest to pursue changes, lobbyists would selectively distribute information that aligns with the beliefs of specific policy-makers.	Focuses on ambiguity, meaning that policy-making is far from an exact science, and that policy expert, politicians and interest groups make policy options go through a selection process that is characterized by incomplete information and financial, technical, and political limitations. Industry lobbyists thus need to provide (mis)information such that only those policy options survive that align with industry interests.	Boundedly rational, emphasizes that individuals are driven by beliefs and prone to “devil shift,” ie, perceiving political opponents as being more powerful and evil than in reality. For example, “Big Industry” is an often-used metaphor used by pro-public health coalitions, and industry lobbyists similarly frame opposing coalitions to have questionable motives and skills.	Homo narrans, identifies that humans shape their ideas through heuristics that include the primacy of affect, groups, identities and recognition/selective exposure. The NPF would thus allow more detailed analysis of industry framing, including how it uses (group) identities, and how this impacts policy processes.
Assumed choice making process of actors	PET requires investigating how actors (eg, interest groups such as industry lobbyists and front groups) attempt to influence policy in different venues (specialized subsystems, eg, Finance and Health departments). They further strategize when and how to target political leaders when an issue gains prominence on the macropolitical agenda. PET does not focus on the specific resources and beliefs that drive the political behaviour of industries, their front groups or other actors.	The MSF emphasizes the role of “policy entrepreneurs,” individuals that actively pursue a specific policy option and try and frame it such that it aligns with how society understand it (problem stream), that is acceptable to politicians (political stream), and that is seen as technically, legally and financially feasible (policy stream). Industry lobbyists can take up this entrepreneurial role or threaten/undermine entrepreneurs that pursue policy contradicting their interests.	ACF centres around the coalition-building and strategic actions of actors, and how they learn through interacting with the political system. It helps understand how industry lobbyists and their front groups learn from losses and wins in policy-making.	NPF assumes that all actors interested in policies strategically use narratives to influence public opinion and policy. It would therefore allow for comparative analysis of how different health-harming industries narrate the public health problem they are interested in, and how these narratives change over time as the issue gains or loses salience.
Assumed role of institutions for decision-making	Assumes that institutional venues and their rules are persistent and stabilizing forces in the policy process, and thus impact the amount of friction towards policy change. Institutions thus play a key role in maintaining or disrupting policy monopolies and in turn maintaining or disrupting policy outputs.	MSF recognizes that institutions play a role in the problem definition, policy development and politics, although (in)formal rules and venues of decision-making are not emphasized. It does recognize explicitly that political institutions in particular can open or close windows of opportunity.	ACF provides less direct guidance on the role of institutions in the policy process as institutions are not its main focus. The framework views institutions as environments with which shape coalitions interact, and that coalitions learn from their failures and successes in this interaction.	NPF sees institutions as part of context within which the narrative about a specific issue resides, but is less focused on the role of institutions in the policy process. This could refer, eg, to how industry lobbyists use fears of “Big Government” in storylines focused on undermining support for regulations.
Networks/ subsystems	PET emphasizes how “policy subsystems” handle policy issues when these are not elevated to the macropolitical agenda. For example, the education department “owns” education policy, unless education temporarily is a high-agenda item requiring the attention of political leaders. Health-harming industry lobbyists therefore need close ties with these subsystems, eg, integrate industry-friendly education materials to education departments, and maintain strong networks with political leaders, in case the harms related to the industry suddenly become a political priority.	MSF emphasizes how policy networks can consist of broad communities that go through policy selection processes together, but has less focus on the interaction of these communities with subsystems that “own” an issue in stable times. Industry lobbyists may seek to influence these networks but the MSF provides little guidance how that relates to formal institutions.	ACF highlights how coalitions interact with formal policy subsystems. Eg, how industry lobbyists learn to alter their advocacy coalitions such that they strategically position parts of their coalition to talk to the right policy subsystems at the right time. Eg, tobacco industry lobbyists with expertise on trade agreements would talk to foreign affairs departments more effectively following increased insight into the institutional rules of the game of establishing trade agreements.	Similar to ACF, NPF describes how coalitions interact with formal policy subsystems, but additionally specifies how this interaction is impacted by “policy regimes”: overarching stories of an issue within a particular policy domain. In obesity, for instance, pharmaceutical companies might fund and coalesce with patient organizations to share stories with health departments that frame obesity as a problem requiring individual treatment.
Ideas or beliefs	Dominant beliefs in PET are that there is a monopoly of understandings in established subsystems; and that new ideas breaking through can trigger policy change. A single, established policy image such as that obesity is the result of individuals making bad choices about diets and exercise would be indicative of the weight-loss industry’s policy monopoly. Change may occur when multiple policy images of “obesity” exist at the same time, explaining why policy battles are often battles over images and language.	MSF places much emphasis on how beliefs in society with agenda prominence shift, and how policy solutions over time become acceptable to a policy community and politicians. Eg, as obesity is increasingly seen as a societal problem, and more polities implement soda taxes, policy-makers and politicians get “used” to the idea of considering a soda tax themselves. Industry lobbyists therefore attempt to impact how society thinks about issues, and which policy solutions gain salience.	Beliefs are at the core of ACF, given that advocacy coalitions by definition share a common belief system, including on core ideological beliefs, policy beliefs around the causes of and broad solutions to a specific issue, and secondary beliefs about preferred policy measures. ACF allows for an analysis of how industry lobbyists organize ideological alignment with their front groups, how they narrate cause and effect theories, and how that leads them to propagate self-regulatory solutions while undermining more effective regulations.	The core focus of NPF is how actors use narrative strategies grounded in normative and empirical beliefs, which are part of cultural norms and values. Industry lobbyists have aggressively promoted an interpretation of “freedom” as negative freedoms (the absence of interference), for instance, to strengthen anti-nanny-stateism stories that block public health regulations.
Assumptions about events	Assumes that events shift the macropolitical agenda and trigger major change. These events are hard to predict and can be endogenous (eg, farmer interest groups organizing highway blockades) or exogenous to the political system (eg, natural disasters). They can also be small (eg, bandwagon effects when media jump unto personal stories) and personal (eg, a politician getting sick). Whether events lead to change depends on the level of organization of actors and their ability to break through institutional friction towards change during brief periods of high attention.	MSF assumes focusing events cause movement in the problem stream, which only triggers change if the policy and political stream are also ready for coupling. Industry lobbyists thus can prevent an event to trigger change that would harm their interests by producing disinformation about, eg, the costs or acceptability of policy solutions.	ACF has less emphasis on events, but does identify how coalitions learn from external events and internal shocks, such as changes in governing coalitions. ACF would thus allow inquiry of how industry lobbyists strategically use external events or internal disputes among their opponents, or how pro-public health coalitions can do the same when events disturb industry coalitions.	The NPF does not explicitly focus on events, although events may help strengthen or break down dominant narratives. Eg, scientific breakthroughs in anti-obesity medication could be used by the food and pharmaceutical industries to strengthen stories that frame obesity as a medical problem of individuals rather than a public health problem caused by modern commercial food systems.

Abbreviations: PET, Punctuated Equilibrium Theory; MSF, Multiple Streams Framework; ACF, Advocacy Coalition Framework; NPF, Narrative Policy Framework.

 None of these theories assume that the individual policy-maker is rational. Whereas PET and the MSF stress that policy-makers ignore most information due to the sheer volume of problems that land on their desks (PET) or the ambiguity of evidence (MSF), the ACF and NPF emphasize that policy-makers are driven by beliefs shaped through narratives (NPF). How *actors*— organizations, networks, individuals, and coalitions—interact with public policy is a basic feature of the policy process and thus part of all four theories. For instance, PET focuses on how interest groups and other lobbyists and advocates interact with formal policy-making institutions such as government departments, and the NPF is interested in how actors strategize public policy discourse. The four theories also configure the role of beliefs and events differently. Whereas PET and MSF have a central role for events, beliefs play a central role in the ACF and NPF. For instance, in PET, long-lasting states of policy inertia can sometimes give way to radical change through a “bandwagon effect” of unforeseen events that change information flows about an issue and thereby increase its salience leading to change in how that issue is understood in public and political arenas (ie, its “policy image”)­­.

 In sum, policy process theories such as the four outlined in Table share an aim to understand how actors, including health-harming industries, seek to influence the way in which the public, politicians and policy-making institutions see an issue, and how these normative and empirical beliefs shape related public policies. The theories share recognition that generating precise predictions for policy change is impossible, but they can outline broad directions for change based on retrospective analysis. What (combination of theories) can best be used in the context of the CDoH depends on the questions asked: eg, PET in relation to policy inertia, NPF in relation to discourse, ACF in relation to coalitions, and MSF in relation to a specific policy.

## Corporate Political Activities Indicative of the Strength of an Industry’s Policy Monopoly

 If we resort back to our earlier observation that corporate-political activities of unhealthy commodity industries are likely to differ across time and space, we assume a central role for institutions, as they set the formal rules in the policy-making process – which closely aligns with PET. PET’s “policy monopoly” concept helps to explain what frames and actions are used when, where and by whom. Under a policy monopoly, stakeholders such as unhealthy commodity industries and their allies try to dominate what is said about an issue (the discourse) and by whom (the players). When industries have successfully created a policy monopoly, there may be no need for defensive actions such as “taking legal action” or “obstructing public health campaigners.”^1^ In such cases, the image of an issue and players involved are so tightly aligned with the industry’s interests, that anti-industry rhetoric is unlikely to gain traction. In a recent analysis of Dutch newspaper coverage of food policies, for instance, we found that food industry actors were only featured in 4.8% of the newspaper articles. Consumers (34.6%) and academics (26.2%) were featured much more often.^17^ When food industry actors were represented, they generally opposed food policies such as food taxes and advertisement bans with conventional framing around paternalism and individual responsibility. This apparent absence of the industry in public discourse is indicative of a successful policy monopoly, as the industry apparently did not even have to publicly defend its interests. Empirical analysis has further shown the impact of the explicit choice of the Dutch government to give the food industry a seat at the table in developing public health nutrition policy: it allowed the food industry to promote self-regulatory policies that mainly target individuals through formal institutional arrangements, and to discredit statutory policies that target populations, with less of a need to pursue tacit actions through informal institutional arrangements.^11^

 When an issue’s policy image shifts, existing policy monopolies change their framing and actions accordingly. For instance, the tobacco industry’s denial that smoking caused lung cancer lost credibility when scientific evidence became irrefutable. The industry shifted towards manufacturing doubt about second-hand smoking, and when that became untenable, it started promoting harm-reduction strategies. The credibility and thus the strength of the tobacco industry’s policy monopoly decreased in due process. According to PET, in the still hypothetical situation where even the industry’s harm reduction strategy is seen as incredible by a majority of the public, the industry’s policy monopoly would crumble further.^16^ Further scholarly work could therefore test which assemblages of corporate political framing and actions by unhealthy commodity industries, issue salience, and maturity of the industry signal a strong, weak, strengthening, or declining policy monopoly.

 That a coherent policy image is indicative of the power of a policy monopoly is something we saw in a study where we used stock-and-flow modelling, PET, and a literature review to comprehend why obesity prevention is “stuck.”^18^ Due to effective framing by the food and weight-loss industries, obesity is seen by many politicians, lay people and health professionals as a matter of individual willpower. And with the availability of weight-loss medication (Glucagon-like peptide 1), obesity is increasingly being seen as a disease, which might^14^ or might not^19^ shift the discourse away from obesity as an epidemic. Public policy has strengthened this framing and these industries’ ownership over “solutions” by focusing on individual behavior, treatment, and dieting. We found that these industries’ power positions can be challenged when stronger advocacy coalitions between people with lived experience and formal policy-making institutions inject more “real-world evidence” into public and political discourse to shift the industry-manufactured image of obesity as an individual problem (reflecting the importance of advocacy coalitions and narratives, see Table). Shifting the discourse requires science and science dissemination that raises awareness around the societal and economic causes and consequences of obesity; that individual-targeted behavioral change interventions do not really work; that policies that address obesogenic environments are feasible and effective; and that de-normalizes the role of industries with vested interests in the obesity epidemic. When these elements effectively challenge the framing of obesity as an individual problem, chance events that briefly propel obesity’s agenda position become more likely to change obesity’s policy image and the policy outputs that flow from this image.^18^ As our analysis used PET, however, it did not give specific insights into anticipating how and when which corporate-political activities will try to disrupt such reframing. Analysing which framing and action strategies of the food and weight-loss industries are anticipative of signal shifts in policy images likely requires theoretical input on disrupting coalitions (ie, ACF) and narrative competition (NPF) – and may generate insight into when and how a policy monopoly may be disrupted.

## Rooting Corporate Political Framing Into Moral Values

 Digging deeper into an issue’s salience and the way in which it is framed and understood, we think that Ulucanlar and colleagues’ framework should also be connected to Lakoff’s work on the metaphoric conceptualization of human cognition.^20^ Lakoff built his theory on neurolinguistic analysis of how people with diverse political preferences think. He found two diametrically opposed archetypical moral frameworks that he labelled “strict father morality” and “nurturant parent morality.” As an example, we link these moral frameworks to the problem of addiction. “Strict fathers” see addiction as a problem of personal values, not social change or commercial exploitation. Their answer is teaching self-discipline, and punishment of those that lack discipline, while government assistance would be immoral as it would lead to dependence. “Nurturant parents” approach addiction as a problem of despair over social conditions and commercial exploitation. Their answers to addiction flow from a focus on empathy and include regulatory protection against social and commercial conditions that drive addiction, and empowerment of people with addiction so they gain dignity and self-nurturance. Unhealthy commodity industries need to deal with governments as strict fathers and nurturant parents, because both archetypical moral frameworks can be at play at the same time or take turns in political contexts. Responses to the CDoH would therefore benefit from investigations of how industries adapt their framing and actions to moral frameworks that differ across political parties whose relative importance changes over time (as suggested in NPF). Also, acknowledging that public health responses to address corporate vectors of diseases are centered squarely in nurturant parent morality,^21^ another promising avenue of research would be to study effective empathy-laden counternarratives.

 All in all, the Ulucanlar model is an excellent tool to analyse the frames and actions that contribute to the policy inertia we observe across many commercial vectors of diseases. In order to take the leap to anticipating industry responses and bringing about change, it needs expansion into broader theory on policy change and framing. Policy process theories such as PET and metaphorical framing could help build theory to analyse which corporate political activities are anticipative of a crumbling policy monopoly of unhealthy commodity industries, as well as how to effectively help speed up this process.

## Disclosure of artificial intelligence (AI) use

 Not applicable.

## Ethical issues

 Not applicable.

## Conflicts of interest

 Authors declare that they have no conflicts of interest.
